# Predictive simulations of postural control: exploring the role of signal noise and neural delays in Parkinson’s disease

**DOI:** 10.1038/s41598-026-45161-5

**Published:** 2026-03-24

**Authors:** Julian Shanbhag, Iris Wechsler, Sophie Fleischmann, Bjoern M. Eskofier, Anne D. Koelewijn, Jürgen Winkler, Sandro Wartzack, Heiko Gassner, Jörg Miehling

**Affiliations:** 1 Engineering Design, Department of Mechanical Engineering, Friedrich-Alexander-Universität Erlangen-Nürnberg, Erlangen, Germany; 2 Machine Learning and Data Analytics Lab, Department Artificial Intelligence in Biomedical Engineering (AIBE), Friedrich-Alexander-Universität Erlangen-Nürnberg, Erlangen, Germany; 3 Chair of Autonomous Systems and Mechatronics, Department of Electrical Engineering, Friedrich-Alexander-Universität Erlangen-Nürnberg, Erlangen, Germany; 4 Department of Molecular Neurology, Universitätsklinikum Erlangen, Friedrich-Alexander-Universität Erlangen-Nürnberg, Erlangen, Germany; 5 Digital Health and Analytics, Fraunhofer Institute for Integrated Circuits IIS, Erlangen, Germany

**Keywords:** Postural control, Parkinson’s disease, Neuromusculoskeletal modeling, Predictive simulation, Biomechanics, Neurology, Neuroscience

## Abstract

Postural instability is one of the key motor symptoms of Parkinson’s disease (PD), which worsens with disease progression, leading to an increased fall risk. The complex internal processes of postural control and the elements causing this instability due to PD are still not fully understood. Predictive neuromusculoskeletal simulations can help to gain insights into internal postural control processes and their correlations, which cannot be measured directly. In this paper, we investigated the influence of increased motor signal noise and neural delays on the postural control of quiet upright standing and the resulting sway parameters using a previously published postural control model following assumptions that higher signal noise and neural delays lead to postural impairments in PD. Simulation results were compared to experimental motion capture data from 31 individuals with PD and 31 age- and sex-matched healthy control participants. We found that both higher signal noise and increased neural delays led to an adapted postural control behavior that can be associated with PD. The variations in sway parameters showed high agreement with the previously measured experimental data and the differences between individuals with and without PD. However, further investigation into additional movement tasks is necessary to strengthen these findings.

## Introduction

Postural control is a complex process by which the body aims to maintain balance through muscle reactions controlled by the central nervous system (CNS). Neurological disorders, such as Parkinson’s disease (PD), can impair postural control. Postural instability is one of the key motor symptoms and a major disabling factor in PD^1^. Postural instability worsens as PD progresses and leads to an increased fall risk^2^ as well as fear of falling, which both affect quality of life^3^. A better understanding of these mechanisms can enable improved diagnostics and therapeutic approaches for individuals with PD.

To maintain balance, the body aims to keep the center of mass (COM) above the base of support. The process of keeping or re-positioning the COM above this area is controlled by the CNS^4^. Generally, the neural circuitry involved in postural control acquires information from several sensory systems (somatosensory, vestibular, and visual system) and centrally processes this information to initiate specific muscle reactions necessary for maintaining or regaining balance^5^. Additionally, this process is subject to internal noise, including signal noise^6,7^, breathing, heartbeat^5^, and neural delays. Neural delays reflect the time required for sensory afferents, central processing pathways, including cortico–basal ganglia circuits, and motor efferents to transmit and transform information relevant for postural control. Transmission delays within the basal ganglia can modulate oscillatory activity in both healthy and Parkinsonian conditions^8,9^. Small increases in these delays can promote or amplify beta-band oscillations, which are higher in PD and can degrade motor and postural control.

In individuals with PD, postural instability leads to increased sway compared to healthy individuals and can be observed in parameters such as an increased sway range or area^10,11^. The underlying internal processes causing this altered postural control behavior in PD are not fully understood. It has been reported that individuals suffering from PD may experience increased motor signal noise^12–14^, which means that these signals are comparatively less accurate. Such alterations of signal noise could account for increased sway parameters, as observed in individuals with PD. Functional changes in the basal ganglia system due to PD could also lead to increased neural processing times of signal information^15^, thus affecting postural control. However, neither motor signal noise nor neural delays can be easily measured directly.

In clinical practice, only parameters or symptoms that are measurable or observable from the outside serve as indicators of the levels of motor control impairments or the progression of rehabilitation interventions. Predictive neuromusculoskeletal simulations can help investigate internal mechanisms of postural control and evaluate changes in internal parameters within the postural control circuitry that cannot be accessed directly through motion measurements alone. The cause-effect relationships of internal model parameters or neural processes and resulting movements can be investigated to deepen our understanding of physiological or pathophysiological mechanisms^16–18^. Such neuromusculoskeletal models and predictive simulations enable the investigation of correlations between internal (experimentally inaccessible) elements and experimentally measurable parameters during postural control. Several approaches already exist to integrate postural control into forward dynamic simulations using biomechanical human models to explore such aspects^19^.

In this paper, we use predictive neuromusculoskeletal simulations to investigate the influence of varying motor signal noise and altered neural delays on postural control. Following literature assumptions^12–14^, we hypothesize that a decreased signal-to-noise ratio, represented by higher noise affecting motor signals, will lead to adapted postural control behavior even in quiet upright standing, comparable to that shown by individuals with PD. This would support the hypothesis that increased noise within the neural circuitry could be an important influencing factor on impaired postural control and instability. Furthermore, we hypothesize that increased neural delays, representing longer processing times due to changes in the basal ganglia system^15^, will also affect postural control in a manner associated with PD. Here, we do not explicitly model circuit-level transmission delays in the basal ganglia system, but approximate their functional effect through a global increase in the total sensorimotor processing time of the control process. Our study aims to elucidate these mechanisms, enhancing our understanding of the relationships between neural dynamics and functional motor outcomes in the context of PD.

## Materials and methods

**Fig. 1:**
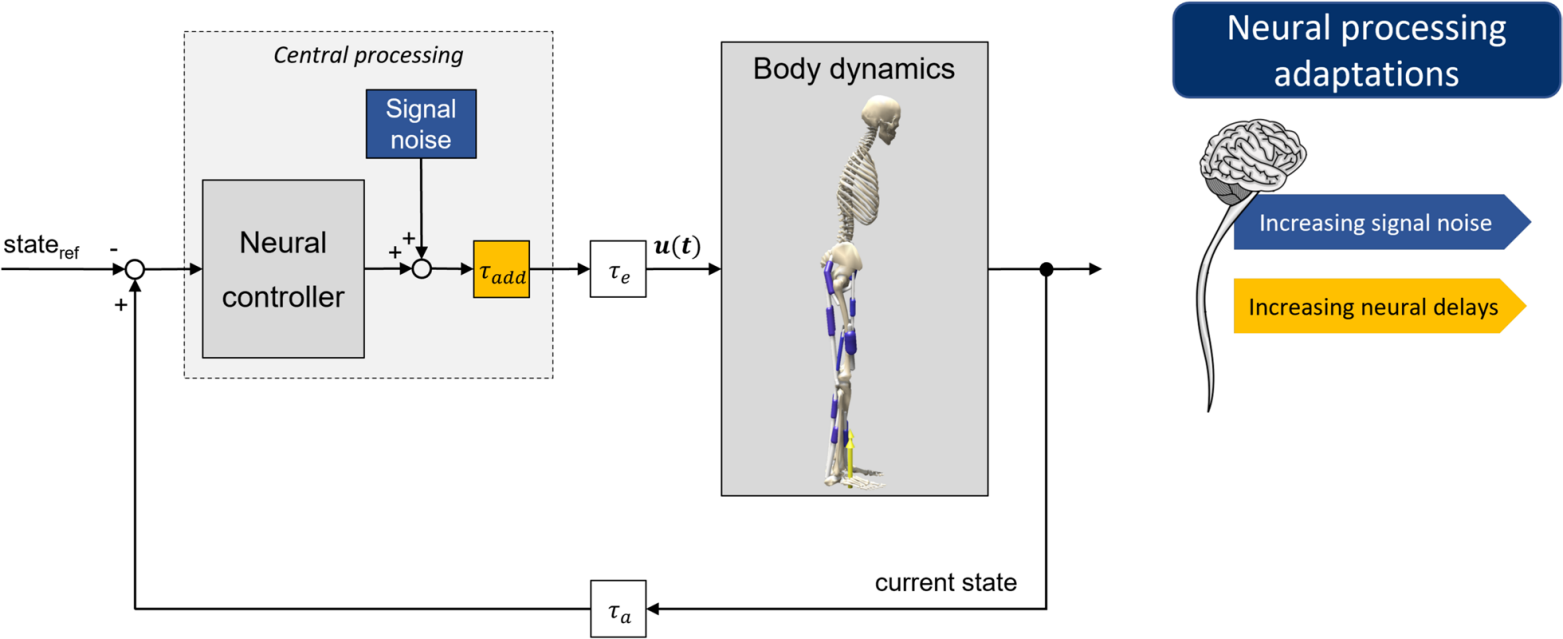
Schematic overview of the postural control model consisting of a musculoskeletal human model with its body dynamics and a neural controller initiating motor signals based on the differences between current and reference states^22^. This process is subject to neural delays (consisting of transmission delays $\tau _a$, $\tau _e$, and processing delays within the neural controller). Furthermore, signal noise (in blue) and an (optional) additional processing delays (in yellow, represented by $\tau _{add}$) are highlighted. They were both adapted to investigate their effects on postural control.

We conducted predictive forward dynamic simulations of postural control using the open-source software SCONE 2.4.0^20^ with Hyfydy^21^. A previously published model^22^, with updates from Shanbhag et al.^23^, served as a starting point. The general model (Section 2.1) and further modifications described in Sections 2.2 and 2.3 are visualized in Fig. 1. The simulation approach is summarized in Section 2.4; experimental data used to evaluate the simulation results, as well as the data evaluation, are described in Sections 2.5 and 2.6.

### Postural control model

We used a musculoskeletal human model according to Delp et al.^24^, with adaptations from Rajagopal et al.^25^, which is distributed with SCONE. The planar sagittal plane model represented nine muscles per leg and resulted in six degrees of freedom (DOFs), because we assumed left-right symmetry. The DOFs included pelvis position and orientation with respect to the ground, as well as the hip, knee, and ankle angles. Sensor information from the somatosensory, vestibular, and visual systems was considered to control posture. For every time step, the current body state, gained from these sensory systems, is compared to a reference state for upright standing. By weighting the differences between these states, a neural controller initiates specific muscle excitations, leading to muscle activations and subsequently muscle forces via Hill-type muscle model dynamics^26^. The postural control circuitry was subject to physiologically plausible neural delays defined in Shanbhag et al.^22^, based on Li et al.^27^, and internal noise representing signal noise and internal perturbations resulting from breathing and heartbeat^23^. The model’s contact between the feet and the ground was represented using two contact spheres at the heel and forefoot positions via Hunt-Crossley forces. Details about this neuromusculoskeletal model of postural control can be found in Shanbhag et al.^22^.

### Signal noise adaptations

We varied the noise ratio of the motor signals to investigate the influence of different signal-to-noise ratios on postural control and resulting biomechanical parameters. Ideal motor signals $u'(t)$, i.e. the muscle excitations sent by the controller to the muscles, were subject to additional noise, resulting in the subsequent signal *u*(*t*). The internal noise was modeled as Gaussian noise.

1 $$\begin{aligned} u(t)&= u'(t) + k_n(t) \cdot R(t) \end{aligned}$$

2 $$\begin{aligned} k_n(t)&= n_c + n_p \cdot u'(t) \end{aligned}$$

The amount of noise was determined by a randomly generated Gaussian number *R*(*t*), which had a mean of 0 and a standard deviation of $k_n(t)$. Further, $k_n(t)$ was defined as a combination of a constant additive component $n_c$ and a signal-dependent proportional component $n_p$ which was multiplied by the ideal muscle excitation $u'(t)$. The constant and signal-dependent noise elements $n_c$ and $n_p$ were both independently incrementally adapted. We selected ranges for both parameters based on the model’s empirical sensitivity to each respective parameter. We investigated changes in $n_c$ ranging from 0 to 3 % of maximal muscle excitations (0.5 % steps), and $n_p$ ranging from 0 to 30 % of the ideal muscle excitations (5 % steps). These ranges were empirically chosen because the sway parameters were only within a range of physiologically plausible values throughout these changes. During the noise adaptations, neural delays were fixed on the constant pre-defined values.

### Neural delay adaptations

In the initial postural control model, neural delays were defined based on the corresponding muscle position as well as the sensory information type. Lumped delays were assumed, consisting of transmission and processing delays, ranging from 25 ms (for somatosensory feedback from upper leg muscles) to 150 ms (for vestibular and visual feedback from lower leg muscles). To investigate the influence of increasing neural delays on postural control, we step-wise adjusted the amount of neural delays (in 5 ms steps) within the range of -25 ms to +100 ms relative to the pre-defined values. We conducted simulations with varying neural delays using two different configurations of internal signal noise: one with $n_c = 0.5$ % and $n_p = 0$ % (lower noise), and the other with $n_c = 1$ % and $n_p = 10$ % (higher noise).

### Simulation approach

The optimization of free parameters and simulation settings were based on those from Shanbhag et al.^22^. We applied single shooting and the pre-implemented covariance matrix adaptation evolution strategy (CMA-ES) algorithm of SCONE^20^ to optimize the simulation scenario. A cost function was used to minimize muscular effort, represented by muscle activations. For further details about the cost function and additional descriptions about the optimization procedure, please refer to Shanbhag et al.^22^. For each simulation scenario, a quiet upright standing task was optimized for a total of 75 seconds, of which we analyzed the last 60 seconds (due to a non-representative pre-defined starting position). The sampling frequency was set to 200 Hz.

### Experimental data

Simulation results for both parameter variations were compared to experimental data from 31 individuals with PD and 31 age- and sex-matched healthy control (HC) participants. The study data were obtained from Shanbhag et al.^23^. The PD group showed mild to moderate motor impairment indicated by Hoehn & Yahr (H&Y) staging ($2.1 \pm 0.6$) and UPDRS-III ($20.8 \pm 8.0$). Characteristics of the study cohort are provided in Table 1. In this study, motion capture data for 60-second quiet upright standing tasks were recorded with an optical motion capture system and two force plates. Recorded marker and force plate data were pre-processed with a third-order Butterworth filter (cutoff frequency 10 Hz) and subsequently evaluated using a three-dimensional musculoskeletal model based on Rajagopal et al.^25^ in OpenSim 4.4^28^. The study was conducted in accordance with the Declaration of Helsinki and approved by the Ethics Committee of the FAU Erlangen-Nürnberg (protocol code 20-473_1-B, date of approval 09 January 2023). All participants have signed a written consent form for this study. Further details about the two participant groups, the data collection, and the data preparation can be found in Shanbhag et al.^23^.

**Table 1:** Characteristics of study cohort, adopted from Shanbhag et al.^23^.

	HC group (n = 31)	PD group (n = 31)	p-value
Sex (male:female)	18:13	25:6	0.0538
Age (years)	61.65 ± 13.39	62.65 ± 12.49	0.7621
Disease duration (years)	–	7.26 ± 5.18	–
H&Y (score)	–	2.13 ± 0.56	–
UPDRS-III (score)	–	20.81 ± 7.97	–
MoCA (score)	26.61 ± 2.40	24.42 ± 4.06	0.0391*
FES-I (score)	17.53 ± 2.66	23.26 ± 7.58	0.0012*

*Significant group difference ($p<0.05$).

### Data evaluation

To evaluate influences of noise ratio and neural delay adaptations on postural control, we determined biomechanical and sway parameters from both simulation and experimental results. Derived parameters were center of pressure (COP) path length, range, position (expressed as the relative location of the COP on the base of support), and frequency, complemented by joint angle ranges of motion (ROMs). Additionally, we assessed the mean muscle activities from the simulation results. Calculations of these parameters were also obtained from Shanbhag et al.^23^. Since the postural control model represents a two-dimensional sagittal plane model, all parameters were limited to anterior-posterior movement elements. In the following, we will use the term *sway parameter* to refer to these parameters in general.

Partial Spearman’s correlation coefficients *r* were computed to assess the relationship between each of the three adapted model parameters – constant noise, proportional noise, and neural delays – and the resulting sway parameters, thereby investigating how much each parameter individually influences the model. For each model parameter, partial correlations were calculated while statistically controlling for the effects of the other two model parameters. Correlations were considered statistically significant if the corresponding p-value was below the Bonferroni-adjusted threshold of $p < 0.0056$, accounting for nine output sway parameters. Effect sizes were determined according to Cohen^29^ (small: $0.1\le r<0.3$, moderate: $0.3\le r<0.5$, strong $0.5\le r\le 1$).

## Results

Experimental data from HC participants and individuals with PD (analyzed in detail in Shanbhag et al.^23^) showed significant group differences in COP path length, COP range, and all joint angle ROMs. No significant differences were observed for COP position and frequency, although a slight forward shift of the COP position was detected in individuals with PD. As both groups were matched for age and sex, the differences were assumed to derive from the influences of PD.

### Influence of increased noise

**Fig. 2:**
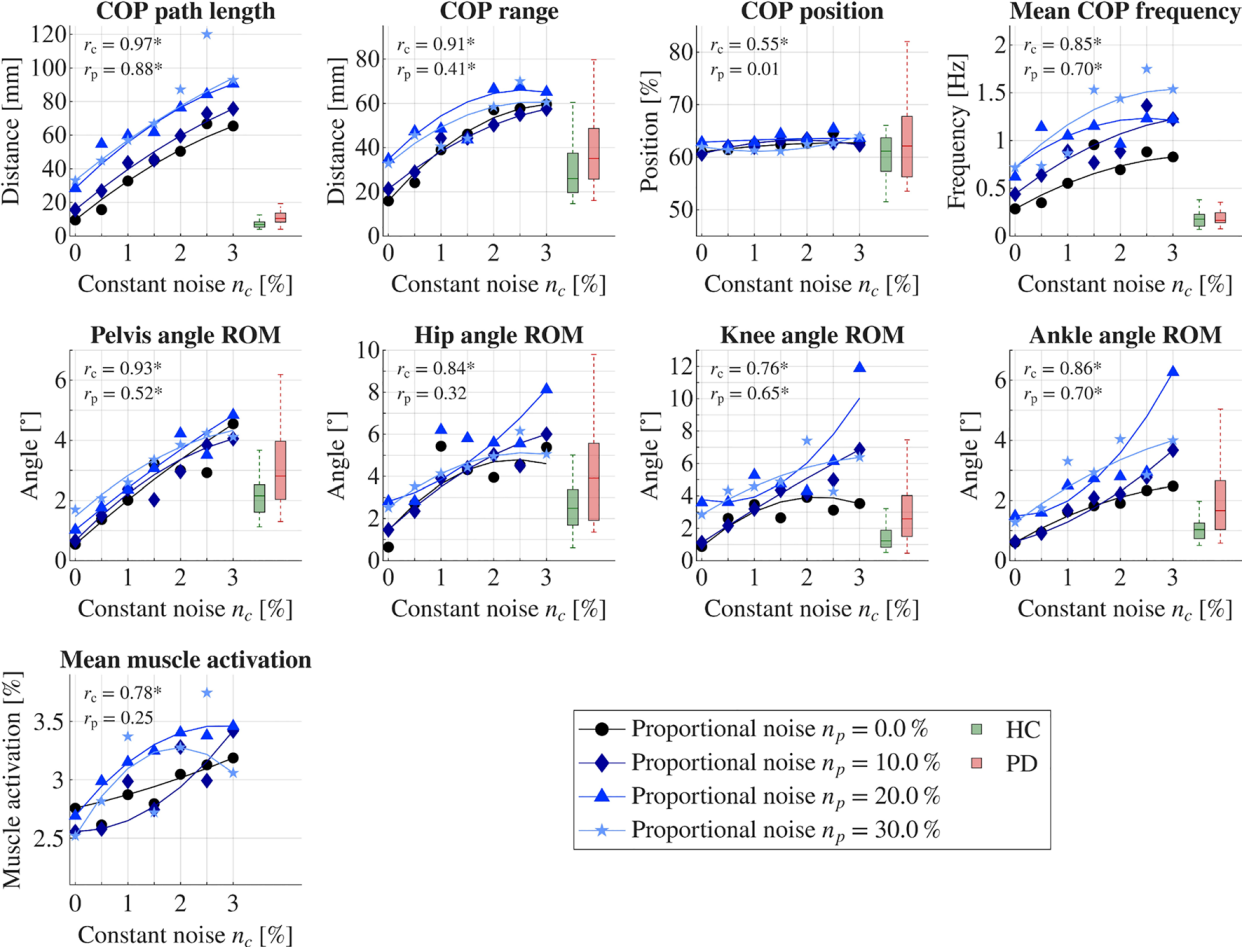
Influence of signal noise on postural control behavior. The simulation results are represented by center of pressure (COP) parameters, joint angle ranges of motion (ROMs), and muscle activations during quiet upright standing depending on altered constant and proportional signal noise. For representative purposes, only the configurations for $n_p=$ 0, 10, 20, and 30 % are shown; additional calculations were performed for $n_p=$ 5, 15, and 25 %. Partial correlations between constant and proportional noise with their respective sway parameters are denoted by $r_c$ and $r_p$, respectively. Experimental data for healthy control (HC) participants (green) and individuals with PD (red) are represented for comparison. No experimental data could be provided for relative muscle activations.

We found that all sway parameters (COP-based parameters as well as joint angle ROMs) resulted in higher values with increased signal noise. Similarly, mean muscle activations increased with higher signal noise. The results of all simulations with adapted signal noise are represented in Fig. 2. This overall increase in resulting sway parameters was evident for both increasing constant ($n_c$) and proportional noise ($n_p$); however, the influence of constant noise showed to be more prominent. Strong correlations ($0.55 \le r_c \le 0.97$) were detected for constant noise and resulting sway parameters, all of them were significant ($p<0.0056$). Medium to strong correlations ($0.32 \le r_p \le 0.87$) were observed for proportional noise and resulting sway parameters except from COP position ($r_p = 0.01$) and mean muscle activation ($r_p = 0.25$); however, not all of the latter correlations were significant (COP position, hip angle ROM, and mean muscle activation did not change significantly). Correlations showed to be smaller for proportional noise compared to constant noise. All correlation coefficients are shown in Fig. 2 and summarized in Table 2. Experimental data of the HC (green) and PD groups (red) were included for comparison. In general, simulated sway parameter trends aligned with our experimental findings and the respective group differences observed between the HC and PD participants.

### Influence of increased neural delays

**Fig. 3:**
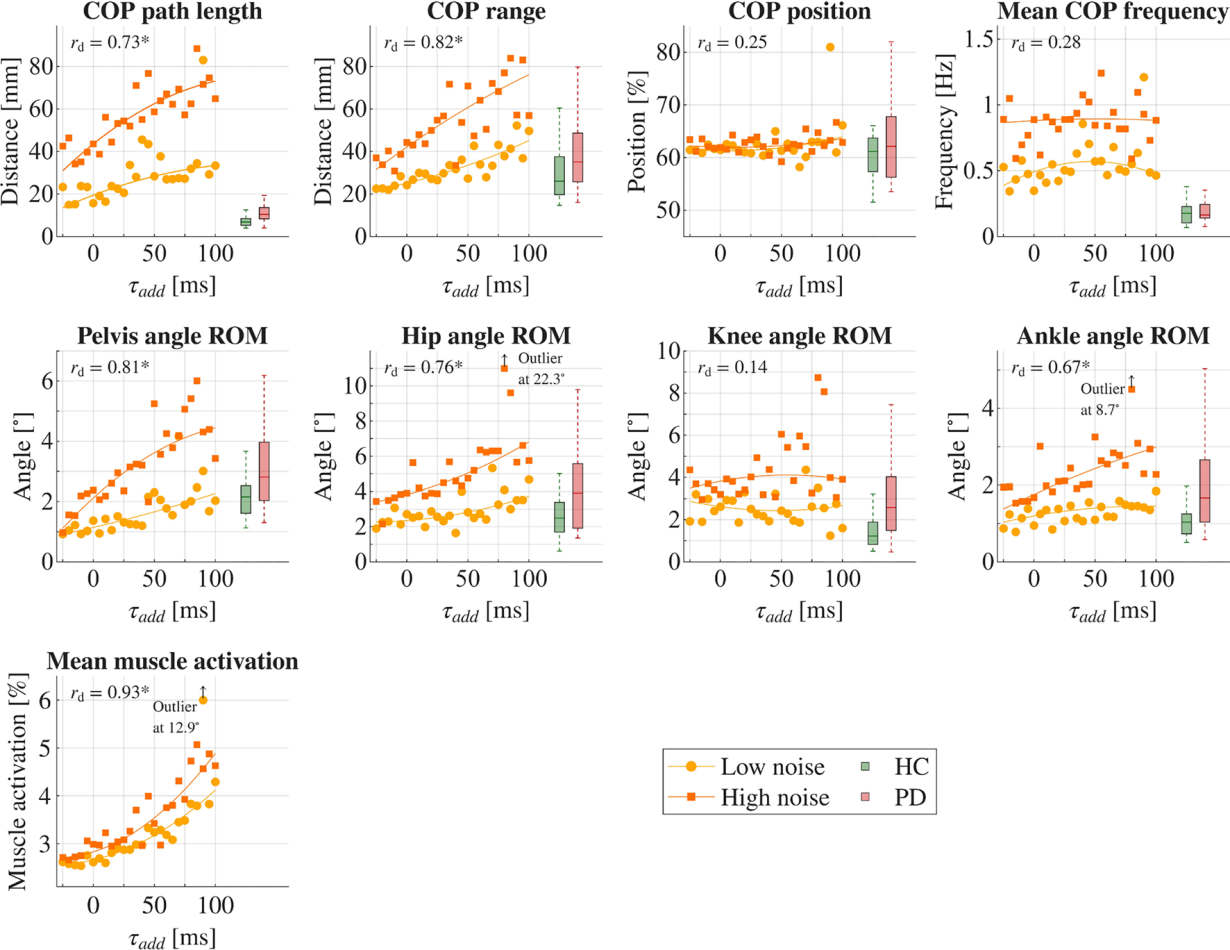
Influence of neural delays on postural control behavior. The simulation results are represented by center of pressure (COP) parameters, joint angle ranges of motion (ROMs), and muscle activations during quiet upright standing with altered neural delays ($\tau _{add}$). Simulations were conducted for two scenarios: one with a small amount of signal noise (0.5 % constant noise, 0 % proportional noise, yellow) and another with a higher amount of signal noise (1 % constant noise, 10 % proportional noise, orange). Partial correlation coefficients are shown for increasing neural delays ($r_d$), controlling for both noise parameters. Experimental data from healthy control (HC) participants (green) and individuals with PD (red) are represented for comparison. No experimental data could be provided for muscle activations.

We found that most sway parameters of simulated postural control increased with additional neural delays. Consistently, significant ($p<0.0056$) strong correlations ($0.67 \le r_{d} \le 0.93$) were observed for increasing neural delays ($\tau _{add}$) and all resulting sway parameters, except for COP position, mean COP frequency, and the knee angle ROM. $r_d$ represents the correlation coefficient for the adapted neural delays and resulting sway parameters, summarizing both noise configurations. Simulation results with additional neural delays are represented in Fig. 3. In this figure, experimental data from HC participants and individuals with PD were again included for comparison, and correlation coefficients are represented. Simulations with lower (yellow) and higher signal noise (orange) resulted in comparable trends for the aforementioned sway parameters. The COP position, COP frequency, and knee angle ROM did not correlate significantly with varied neural delays, and showed only small correlation coefficients ($0.14 \le r_{d} \le 0.28$). Mean muscle activations increased significantly with higher neural delays for both noise configurations. Correlation coefficients are summarized in Tab. 2. Again, the overall trends in simulated sway parameters reflected experimental data and group differences between HC and PD.

**Table 2:** Partial correlation coefficients *r* and p-values for the associations between motor signal noise (constant noise $n_c$ and proportional noise $n_p$) and sway parameters, as well as between neural delays ($\tau _d$) and sway parameters used to characterize postural control behavior.

	Constant noise $n_c$		Proportional noise $n_p$		Neural delays $\tau _d$	
Parameter	$r_c$	$p_c$	$r_p$	$p_p$	$r_d$	$p_d$
COP path length	0.97	< 0.001*	0.88	< 0.001*	0.73	< 0.001*
COP range	0.91	< 0.001*	0.41	0.004*	0.82	< 0.001*
COP position	0.55	< 0.001*	0.01	0.96	0.25	0.08
Mean COP frequency	0.85	< 0.001*	0.70	< 0.001*	0.28	0.05
Pelvis angle ROM	0.93	< 0.001*	0.52	< 0.001*	0.81	< 0.001*
Hip angle ROM	0.84	< 0.001*	0.32	0.03	0.76	< 0.001*
Knee angle ROM	0.76	< 0.001*	0.65	< 0.001*	0.14	0.34
Ankle angle ROM	0.86	< 0.001*	0.70	< 0.001*	0.67	< 0.001*
Mean muscle activation	0.78	< 0.001*	0.25	0.08	0.93	< 0.001*

*Significant correlation for Bonferroni-adjusted threshold.

## Discussion

We investigated the influence of signal noise and neural delays on postural control and the resulting sway parameters. Through two simulation approaches, we incrementally increased signal noise in one and neural delays in the other, subsequently analyzing the sway parameters. For both simulation approaches – alterations in signal-to-noise ratios and neural delays – we observed changes in the resulting sway parameters. These changes generally showed strong correlations between the altered internal model parameters (signal noise or neural delays) and the predicted sway parameters. The directions of the resulting sway parameter progressions were in line with experimental data and the observed trends between participants with and without PD, supporting the suggestion that individuals with PD may exhibit increased noise levels and larger neural delays as a result of their disease. The simulation outcomes provided promising insights into the effects of internal neural parameters on postural control and sway parameters. Although we could only compare simulation results and experimental data qualitatively (Figs. 2 and 3), a clear tendency of simulation results aligning with experimental data could be detected. The HC group showed a better fit for lower-noise simulation results and lower neural delays, whereas the PD group corresponded more closely with higher-noise simulation results and increased neural delays. The only low (and not significant) correlation coefficients observed in simulation scenarios of adapted neural delays pertained to the COP position, mean COP frequency, and knee angle ROM. Notably, the former two parameters were also the ones that did not exhibit significant differences in the experimental data between the HC and PD groups. Therefore, it is reasonable that we did not observe significant changes in our simulation results, as such changes were not anticipated based on our parameter variations intended to reflect PD behavior.

Variations in signal noise, especially constant noise variations, considerably affected the resulting sway parameters. The effect of proportional noise was smaller than constant noise, but still moderate to high. However, we hypothesize that the effects of proportional noise will gain importance in movement tasks that require higher muscle activations and reactive motion behavior compared to the current simulations of quiet upright standing. In such cases, the amplitude-dependent nature of proportional noise will increase its effects on muscle excitation signals, particularly during movements that demand higher muscle activations.

Although our findings demonstrated PD-associated postural control behavior, and many alterations in sway parameters of experimental data could be explained by increased signal noise and neural delays in our simulations, other or additional factors may contribute to postural control, leading to increased postural instability in PD. As the investigated elements of signal noise and neural (processing) delays represent internal body parameters, it is currently almost impossible to finally verify these aspects as the causes of the described postural control alterations. However, to further support our findings, additional movement tasks such as perturbed upright standing, walking, or transfer tasks could be investigated to determine whether similar effects can be observed.

Our simulation results still exhibited systematically higher COP path lengths and frequencies compared to experimental data. One possible interpretation is that the signal noise could consist of other frequency components rather than being Gaussian distributed, based on a 200 Hz sampling frequency, as in our simulations. Therefore, it would be beneficial to model additional noise configurations, such as colored noise with different frequency bands, and to compare different noise insertion points. However, even the results of the noise-free simulations lie at the upper limits of COP path length and COP frequency. Another possible reason for higher sway frequencies could be the modeling of the model’s ground contact, which is often simplified in predictive simulations^30^. However, this aspect can be crucial for obtaining accurate simulation results^31^. Kinematics and COP progression can be affected by inaccurate foot-ground contact models^31^. Additionally, the current model applies continuous control. It is still debated in the literature whether maintaining balance is a continuous or intermittent control process. There are also examples of intermittent control models that have successfully simulated PD-like postural control behavior^32,33^. Further research is necessary to address these aspects.

In our current model, we introduced a simplified, aggregate increase in neural delays to investigate how increased sensorimotor processing time could affect postural control at the behavioral level. In a next step, a more detailed basal ganglia model could be incorporated to extend the current framework and even better represent internal neuromechanical processes and changes due to PD. Since we have shown that the model adjusts its postural control when neural delays are altered, these delay changes could be simulated more specifically within a basal ganglia framework.

Our current model represents DOFs and movements in the sagittal plane. However, important elements of postural instability due to PD can also be observed in the frontal and coronal planes^34^. A model extended to three-dimensional movements could further increase its sensitivity to the parameter adaptations applied in this paper and capture more complex interactions between compensation strategies occurring across different movement planes, including those in the frontal plane.

So far, we could not compare simulated muscle activations to experimental electromyography (EMG) data directly, as participants of the previous study^23^ did not perform maximum voluntary contraction measurements before EMG data were collected. Therefore, it was not possible to obtain absolute muscle activation levels. Assessing such additional data in a next step would improve model validation and could enable a deeper analysis of postural control adaptations due to changing sensorimotor model parameters.

In both approaches – adapting signal noise and neural delays – investigating their influence on postural control behavior, the parameters from the other approach were fixed at previously chosen specific values to isolate each respective effect on the resulting sway parameters. Therefore, the simulation results and detected influences due to parameter changes are of an exemplary nature. In a next step, combining both parameters – signal noise and neural delays – in form of a systematic sensitivity analysis could provide even more comprehensive insights into the interaction between these two aspects and their overall influence on postural control. This would enable the classification of experimental data from HC and PD participants within the spectrum of simulation results and the identification of best-fitting parameter combinations for these individuals. From this perspective, the potentials of estimating internal body parameters based on measured movements can become feasible.

## Conclusion

In this paper, we investigated the influence of increased signal noise and neural delays on postural control during quiet upright standing using predictive neuromusculoskeletal simulations. Our findings demonstrated that both parameter adaptations led to increased sway parameters, comparable to experimental data and the tendencies of observed changes associated with PD. Simulations involving additional movement tasks should be explored to further strengthen the findings of this paper. As a next step, experimental data from HC and PD participants could be used to estimate internal parameters that best characterize specific target groups or even individuals, thereby deepening our understanding of internal postural control processes. Prospectively, this approach would enable the classification of experimental data, as well as simulation-based diagnostics and evaluations of rehabilitation interventions by facilitating pre- and post-comparisons based on the experimental data.

## Data Availability

The datasets generated during and/or analyzed during the current study are available from the corresponding author on reasonable request.

## References

[1] Wuehr, M. et al. Effects of low-intensity vestibular noise stimulation on postural instability in patients with Parkinson’s disease. *J. Parkinson’s Disease* **12**, 1611–1618. 10.3233/JPD-213127 (2022). 35491798 10.3233/JPD-213127

[2] Park, J.-H., Kang, Y.-J. & Horak, F. B. What is wrong with balance in Parkinson’s disease?. *J. Movement Disord.* **8**, 109–114. 10.14802/jmd.15018 (2015). 10.14802/jmd.15018 PMC4572660 26413237

[3] Fasano, A., Canning, C. G., Hausdorff, J. M., Lord, S. & Rochester, L. Falls in Parkinson’s disease: A complex and evolving picture. *Movement Disord.* **32**, 1524–1536. 10.1002/mds.27195 (2017). 29067726 10.1002/mds.27195

[4] Winter, D. Human balance and posture control during standing and walking. *Gait Posture* **3**, 193–214. 10.1016/0966-6362(96)82849-9 (1995).

[5] Forbes, P. A., Chen, A. & Blouin, J.-S. Sensorimotor control of standing balance. *Handbook Clin. Neurol.* **159**, 61–83. 10.1016/B978-0-444-63916-5.00004-5 (2018). 10.1016/B978-0-444-63916-5.00004-5 30482333

[6] Todorov, E. & Jordan, M. I. Optimal feedback control as a theory of motor coordination. *Nat. Neurosci.* **5**, 1226–1235. 10.1038/nn963 (2002). 12404008 10.1038/nn963

[7] Harris, C. M. & Wolpert, D. M. Signal-dependent noise determines motor planning. *Nature* **394**, 780–784. 10.1038/29528 (1998). 9723616 10.1038/29528

[8] Liénard, J. F., Aubin, L., Cos, I. & Girard, B. Estimation of the transmission delays in the basal ganglia of the macaque monkey and subsequent predictions about oscillatory activity under dopamine depletion. *Eur. J. Neurosci.* **59**, 1657–1680. 10.1111/ejn.16271 (2024). 38414108 10.1111/ejn.16271

[9] Asadi, A., Madadi Asl, M., Valizadeh, A. & Perc, M. Dynamics of parkinsonian oscillations mediated by transmission delays in a mean-field model of the basal ganglia. *Front. Cellular Neurosci.* **18**, 1344149. 10.3389/fncel.2024.1344149 (2024). 10.3389/fncel.2024.1344149 PMC10972955 38550919

[10] Błaszczyk, J. W., Orawiec, R., Duda-Kłodowska, D. & Opala, G. Assessment of postural instability in patients with Parkinson’s disease. *Exp. Brain Res.* **183**, 107–114. 10.1007/s00221-007-1024-y (2007). 17609881 10.1007/s00221-007-1024-y

[11] Doná, F. et al. Changes in postural control in patients with Parkinson’s disease: A posturographic study. *Physiotherapy* **102**, 272–279. 10.1016/j.physio.2015.08.009 (2016). 26582134 10.1016/j.physio.2015.08.009

[12] Maurer, C., Mergner, T. & Peterka, R. Abnormal resonance behavior of the postural control loop in Parkinson’s disease. *Exp. Brain Res.*10.1007/s00221-004-1852-y (2004). 15007581 10.1007/s00221-004-1852-y

[13] Nogueira, S., Ferreira, E., Geisinger, D., San Román, C. & Suarez, H. Model of postural control system applied in Parkinson’s disease patients. In *2010 Annual International Conference of the IEEE Engineering in Medicine and Biology*, 5452–5455, 10.1109/IEMBS.2010.5626509 (IEEE, Buenos Aires, 2010). 10.1109/IEMBS.2010.5626509 21096282

[14] Permezel, F., Alty, J., Harding, I. H. & Thyagarajan, D. Brain networks involved in sensory perception in Parkinson’s disease: A scoping review. *Brain Sci.* **13**, 1552. 10.3390/brainsci13111552 (2023). 38002513 10.3390/brainsci13111552 PMC10669548

[15] Stelmach, G. E., Teasdale, N. & Phillips, J. Response initiation delays in Parkinson’s disease patients. *Human Movement Sci.* **11**, 37–45. 10.1016/0167-9457(92)90048-G (1992).

[16] Barati, H., Nazari, K., Kardan, I. & Akbarzadeh, A. Predictive Simulation of Hemiparetic Gait Based on Neural Impairments. In *2023 11th RSI International Conference on Robotics and Mechatronics (ICRoM)*, 371–376, 10.1109/ICRoM60803.2023.10412490 (IEEE, Tehran, Iran, Islamic Republic of, 2023).

[17] Van Der Kruk, E. & Geijtenbeek, T. Is increased trunk flexion in standing up related to muscle weakness or pain avoidance in individuals with unilateral knee pain; a simulation study. *Front. Bioeng. Biotechnol.* **12**, 1346365. 10.3389/fbioe.2024.1346365 (2024). 38659645 10.3389/fbioe.2024.1346365 PMC11039967

[18] Veerkamp, K. *et al.* Evaluating cost function criteria in predicting healthy gait. *J. Biomech*. **123**, 110530, 10.1016/j.jbiomech.2021.110530 (2021). Num Pages: 16. 10.1016/j.jbiomech.2021.110530 34034014

[19] Shanbhag, J. et al. Methods for integrating postural control into biomechanical human simulations: A systematic review. *J. Neuroeng. Rehabilit.* **20**, 111. 10.1186/s12984-023-01235-3 (2023). 10.1186/s12984-023-01235-3 PMC10440942 37605197

[20] Geijtenbeek, T. SCONE: Open source software for predictive simulation of biological motion. *J. Open Source Softw.* **4**, 1421. 10.21105/joss.01421 (2019).

[21] Geijtenbeek, T. The Hyfydy Simulation Software (2021).

[22] Shanbhag, J. et al. A sensorimotor enhanced neuromusculoskeletal model for simulating postural control of upright standing. *Front. Neurosci.* **18**, 1393749. 10.3389/fnins.2024.1393749 (2024). 38812972 10.3389/fnins.2024.1393749 PMC11133552

[23] Shanbhag, J. et al. Does reduced reactivity explain altered postural control in Parkinson’s disease? A predictive simulation study. 10.1101/2025.04.30.651447 (2025).

[24] Delp, S. L. et al. An interactive graphics-based model of the lower extremity to study orthopaedic surgical procedures. *IEEE Trans. Bio-med. Eng.* **37**, 757–67. 10.1109/10.102791 (1990). 10.1109/10.102791 2210784

[25] Rajagopal, A. et al. Full-body musculoskeletal model for muscle-driven simulation of human gait. *IEEE Trans. Biomed. Eng.* **63**, 2068–2079. 10.1109/TBME.2016.2586891 (2016). 27392337 10.1109/TBME.2016.2586891 PMC5507211

[26] Millard, M., Uchida, T., Seth, A. & Delp, S. L. Flexing computational muscle: Modeling and simulation of musculotendon dynamics. *J. Biomech. Eng.* **135**, 021005. 10.1115/1.4023390 (2013). 23445050 10.1115/1.4023390 PMC3705831

[27] Li, Y., Levine, W. S. & Loeb, G. E. A two-joint human posture control model with realistic neural delays. *IEEE Trans. Neural Syst. Rehabilit. Eng.* **20**, 738–48. 10.1109/TNSRE.2012.2199333 (2012). 10.1109/TNSRE.2012.2199333 22692939

[28] Seth, A. et al. OpenSim: Simulating musculoskeletal dynamics and neuromuscular control to study human and animal movement. *PLoS Comput. Biol.* **14**, 1006223. 10.1371/journal.pcbi.1006223 (2018). 10.1371/journal.pcbi.1006223 PMC6061994 30048444

[29] Cohen, J. *Statistical power analysis for the behavioral sciences* (Psychology Press, New York, NY, 1988), 2. ed., reprint edn.

[30] Denayer, M. *et al.* A PRISMA systematic review through time on predictive musculoskeletal simulations. *Journal of NeuroEngineering and Rehabilitation* **22**, 10.1186/s12984-025-01686-w (2025). Publisher: Springer Science and Business Media LLC. 10.1186/s12984-025-01686-w PMC12228224 40615923

[31] Millard, M. & Mombaur, K. A Quick Turn of Foot: Rigid Foot-Ground Contact Models for Human Motion Prediction. *Frontiers in Neurorobotics* **13**, 10.3389/fnbot.2019.00062 (2019). Publisher: Frontiers Media SA. 10.3389/fnbot.2019.00062 PMC6693511 31440154

[32] Dash, R., Shah, V. V. & Palanthandalam-Madapusi, H. J. Explaining Parkinsonian postural sway variabilities using intermittent control theory. *Journal of biomechanics* **105**, 109791, 10.1016/j.jbiomech.2020.109791 (2020). Num Pages: 8. 10.1016/j.jbiomech.2020.109791 32423540

[33] Bao, W. & Chen, K. Explaining Parkinsonian postural instability using an improved intermittent control model. *Chaos Solitons Fractals* **182**, 114844. 10.1016/j.chaos.2024.114844 (2024).

[34] Mancini, M. et al. Postural sway as a marker of progression in Parkinson’s disease: A pilot longitudinal study. *Gait Posture* **36**, 471–476. 10.1016/j.gaitpost.2012.04.010 (2012). 22750016 10.1016/j.gaitpost.2012.04.010 PMC3894847

